# Effect of two commercial acellular dermal scaffolds on biological behavior of human gingival fibroblasts

**DOI:** 10.34172/japid.2020.011

**Published:** 2020-07-05

**Authors:** Omid Moghaddas, Behdokht Miremadi, Ehsan Seyed Jafari

**Affiliations:** ^1^Department of Periodontics, Faculty of Dentistry, Tehran Medical Sciences Islamic Azad University, Tehran, Iran; ^2^Private Practice, Tehran, Iran; ^3^Department of Nanotechnology, Faculty of Medicine, Tehran University of Medical Sciences, Tehran, Iran

**Keywords:** Acellular dermis, Cell survival, Fibroblasts, Tissue scaffolds

## Abstract

**Background:**

Periodontal regeneration is an essential goal of periodontal therapy. Acellular dermal matrix (ADM) has been recommended as an alternative to autogenous grafts. However, since it is devoid of cells and vasculature, there are concerns regarding the biological behavior of cells on ADM. This study aimed to assess the effects of two commonly used ADMs on biological behavior, i.e., attachment and proliferation, of human gingival fibroblasts (HGFs).

**Methods:**

This in vitro, experimental study was conducted on explanted and cultured HGFs. ADM types 1 and 2 (n=26; measuring 10×15 mm) were rinsed with saline solution, adapted to the bottom of 52 wells, exposed to HGFs with a cell density of 16,000 cells/mL, and incubated at 37°C for 12, 24, and 84 hours and seven days. Cell attachment was assessed 12 hours after incubation using 4›,6-diamidino-2-phenylindole (DAPI) and methyl-thiazol-diphenyl-tetrazolium (MTT) assay under a fluorescence microscope. Cell viability was assessed at 24 and 84 hours and one week using the MTT assay. Cells were then platinum-coated, and their morphology was evaluated under a scanning electron microscope (SEM). Data were analyzed using ANOVA.

**Results:**

HGFs were evaluated in 60 samples in three groups (n=20). Cell attachment was the same in the three groups, as shown by the MTT assay and DAPI test (P=0.6). Cell viability at one week was 3.73±0.02, 2.88±0.29, and 2.13±0.24 in the control, ADM 1, and ADM 2 groups, respectively. The difference was statistically significant (P=0.01).

**Conclusion:**

Both scaffolds were the same in terms of attachment of HGFs. However, ADM 1 was superior to ADM2 in terms of cell viability and morphology at one week. It was concluded that the quality of acellular dermal scaffolds could significantly influence cellular behaviors and tissue maturation.

## Introduction

Periodontal therapy is performed to provide suitable conditions for the regeneration of the injured or lost periodontium.^1^ If left untreated, gingival recession can cause an unesthetic appearance, root caries, and tooth hypersensitivity.^2,3^ Since the introduction of free connective tissue grafts in 1902 for the coverage of denuded root surfaces,^4^ several techniques, such as the pedicle flap, coronally positioned flap, tunnel technique, bi-papillary flap, rotated palatal pedicle flap, cell sheet engineering, growth factors, stem cells, enamel matrix proteins, guided tissue regeneration, and allografts have been used for this purpose.^5^ Despite excellent esthetic results, all these techniques have high technical sensitivity and limitations in terms of the amount of available tissue, maintenance of clinical properties, and donor site morbidity.^6^

Acellular dermal matrix (ADM) was introduced as an alternative to autogenous graft for periodontal use.^7^ However, the absence of cells and vasculature in ADM decreases tissue compatibility with ADM grafts compared to connective tissue grafts. Autografts enhance angiogenesis due to the presence of vascular anastomosis in the graft. However, allografts are acellular and devoid of blood vessels, and therefore, they depend on host cells and vasculature for reorganization.^8^

Considering the significance of angiogenesis and the availability of different tissue scaffolds on the market, there are several unanswered questions regarding the acceptable attachment and viability of fibroblasts on different commercially available scaffolds. Many in vitro and in vivo studies have evaluated the biological behavior of fibroblasts on different tissue scaffolds. However, studies on the efficacy of ADMs produced in Iran are scarce.

Considering the gap of information in this respect, this study aimed to assess the biological behavior of fibroblasts in terms of attachment and viability on different scaffolds to see if the quality of scaffolds can have any significant effect on it.

## Methods

The present in vitro experimental study was performed on two different ADMs: ADM1, Hamanandsaz Kish Tissue Bank, and ADM2, Iranian Tissue Bank. Twenty-six samples of each ADM scaffold were selected, and twenty-six empty wells served as the control group.

### Cell isolation and culture

Human gingival fibroblasts (HGFs) isolated from the gingiva using the explant method were seeded and cultured. Tissue samples were immediately immersed in sterile phosphate-buffered saline (PBS) (Sigma, USA) supplemented with 100 U/mL of penicillin (Sigma, USA) and 100 µg/mL of streptomycin (Sigma, USA) and incubated at 4°C. The tissue was de-epithelialized and diced into 1–2 mm^2^ segments and immersed in 0.2-µm alpha-minimum essential medium (alpha-MEM; Sigma, USA) consisting of 1 mg/mL of dispace (Sigma, USA) and 2 mg/mL of type IV collagenase (Sigma, USA) and incubated at 37°C for 30 minutes. After preparing the first cell suspension, the tissues were incubated in the same solution at 37°C for 90 minutes. The cell suspension was filtered using 70-µm filter paper and cultured in 75-cm^2^ cell flask (SPL, Korea) containing alpha-MEM supplemented with 15% fetal bovine serum, 100 U/mL of penicillin, 100 µg/mL of streptomycin, 200 mM of L-glutamine, and 10 mM of ascorbic acid bi-phosphate (Sigma, USA). The cells were incubated at 37°C, 5% CO2, and 95% air. After 24 hours, unattached cells were rinsed with PBS, and the medium was replaced every three days.

### Preparation of scaffolds and cell culture

**Figure 1:**
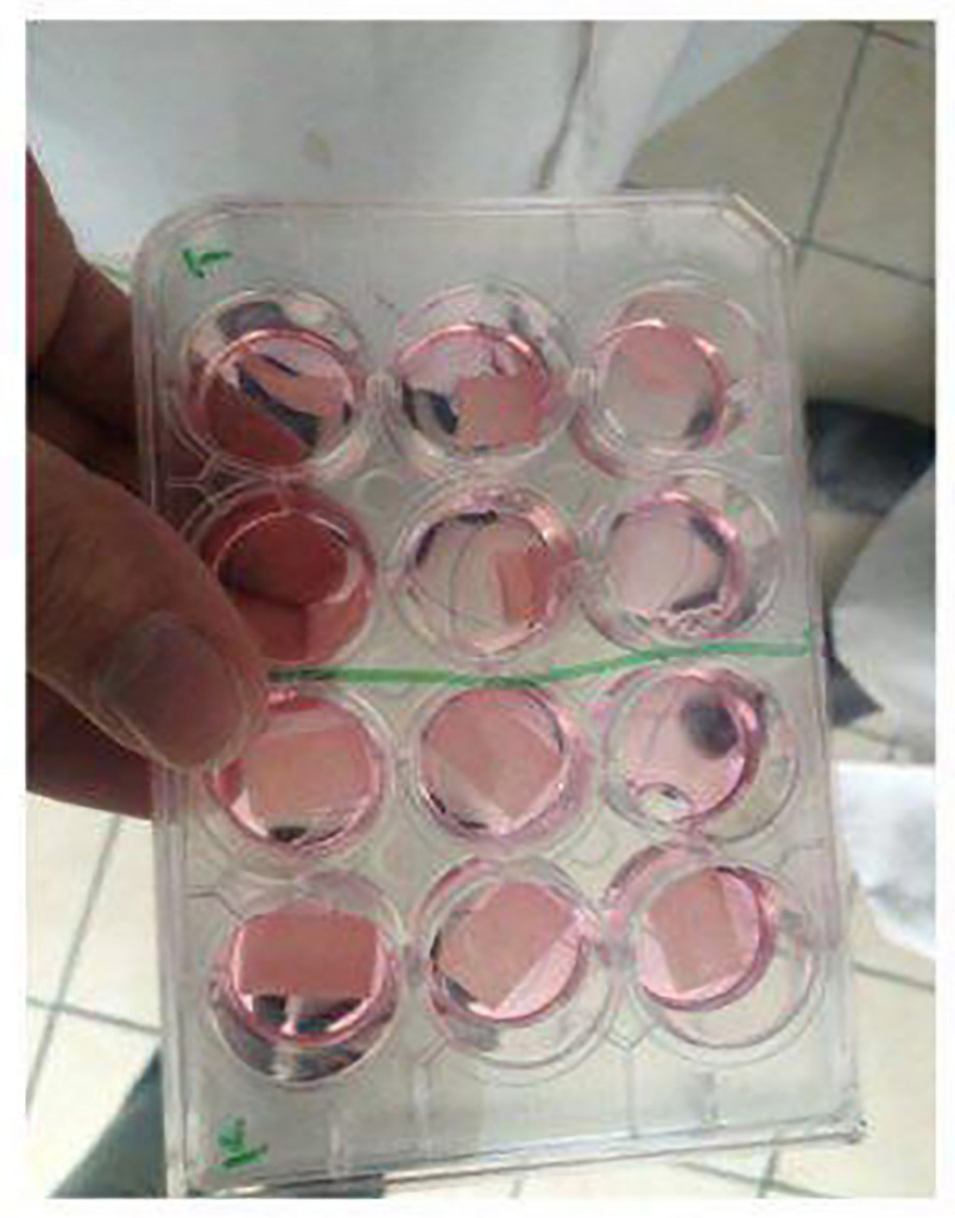
ADM adapted to the bottom of wells.

According to the manufacturers’ instructions, ADM types 1 and 2 were rinsed with sterile saline solution (Sigma, USA) in 50-mL flasks (SPL, Korea) for 10 minutes. They were then cut into 26 rectangular pieces measuring 1×1.5 cm and adapted to the bottom of 52 wells in six plates (SPL, Korea) (Figure 1). In each plate, ADM type 1 was placed in five wells, and ADM type 2 was placed in five other wells. Five empty wells (no scaffold) served as controls. One plate was used for the assessment of cell morphology and contained one well of each type of scaffold and one empty well as controls. Cells with 16,000 cells/mL density were added to the scaffold and control wells, and the plates were incubated for 12, 24, and 84 hours and one week at 37°C and 5% CO2.

Three plates were used for the assessment of cell viability using the MTT assay, and two plates were used for the assessment of cell attachment using 4’,6-diamidino-2-phenylindole (DAPI) and methyl-thiazol-diphenyl-tetrazolium (MTT) assay. Each plate was removed from the incubator at the designated time interval for assessment.

### Evaluation of cell attachment

**Figure 2:**
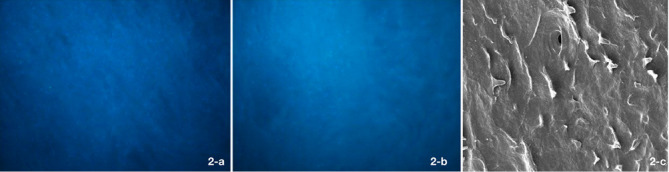
Epifluorescence view of human fibroblasts in the control (A), ADM1 (B), and ADM2 (C) groups stained with DAPI at 12 hours.

After 12 hours of incubation, the cells were fixed with 2.5% glutaraldehyde (Sigma, USA) and stained with 50 µg/mL of DAPI for 30 minutes. They were then rinsed with PBS to remove unattached cells. The cells were then inspected under a fluorescence scanning electron microscope (SEM; Nikon, Tokyo, Japan) at 290 nm wavelength (Figure 2). The cells were counted in five points (four points at the corners and one at the center).^9^ The MTT assay was used to determine the optical density (OD) 12 hours after cell culture to determine the primary attachment of cells to the scaffold and control wells with five repetitions. A standard curve was used to convert OD values to cell counts.

### Assessment of cell viability and proliferation

The viability and proliferation of cells were assessed at 24 and 84 hours, and one week after the incubation using the MTT assay.^10^ This test assesses the activity of mitochondrial dehydrogenase enzyme, which converts the yellow MTT salt to purple formazan crystals. The OD is then determined.^11^ The cell counts in different scaffold groups showed their proliferation capacity and attachment to the scaffolds.^12^ For this purpose, 200 µ of RPMI1640 (Sigma, USA) and 20 µ of MTT (5 mg/mL) (Sigma, USA) were added to the wells and incubated at 37°C and 5% CO2 for four hours.^11^ The absorbance of formazan crystals directly revealed the number of attached cells.^13^ The viability of cells was determined according to a linear diagram indicating the correlation between OD and cell concentration. Measurements were repeated five times.

### Assessment of cell morphology

The cells were seeded onto scaffolds and incubated for 24 hours to assess cell morphology. The cells were then rinsed with PBS twice and fixed in 2.5% glutaraldehyde (Sigma, USA) at room temperature for one hour. The cells were then dehydrated in 50%, 60%, 70%, 80%, 90%, and 100% ethanol and hexamethyldisilazane (Sigma, USA). The samples were then platinum-coated and observed under an SEM (Nikon, Tokyo, Japan). Two parameters were assessed: the surface area of the scaffold covered with cells (in square micrometers) and the sphericity of cells (the ratio of the smaller-to-larger diameter of each cell; elongated cells were scored 0, and spherical cells were scored at 1 because fibroblast cells are spindle-shaped when normal and active, and round when inactive.^9,14^ One sample of each scaffold was scanned under the microscope.

### Statistical analysis

The Mann-Whitney U test was used to assess cell attachment. ANOVA was used to analyze cell viability, and in case of significant difference, a post hoc test was applied.

## Results

### Cell Attachment

Cell attachment was assessed in 24 samples in three groups of control, ADM1, and ADM2, using DAPI and MTT assays. Table 1 shows the attachment rate in the three groups, according to DAPI and MTT assay.

**Table 1:** Cell attachment in the three groups according to DAPI and MTT assay

**Group/Technique**	**DAPI**	**MTT**
**Control**	221.6±25.28	0.25±0.031
**ADM1**	208.3±23.71	0.24±0.032
**ADM2**	217.1±46.19	0.21±0.017

In DAPI test evaluation, the highest cell count was noted in the control group, with the lowest in the ADM1 group. According to ANOVA, the difference in this regard was not significant among the three groups (P=0.6).

In the MTT assay, the highest cell count was noted in the control group, with the lowest in the ADM2 group. According to ANOVA, the differences in this regard were not significant among the three groups (P=0.6).

### Cell Viability

Assessment of cell viability was carried out on 36 samples in the three groups of control, ADM1, and ADM2, using the MTT assay. Table 2 shows cell viability in the three groups at different time intervals.

**Table 2:** Cell viability(CV) in the three groups at different time points

		**Viability rate**	** CV**
**Control**	24 hours	0.31±0.02	6
	84 hours	1.04±0.104	10
	One week	3.73±0.2	5
**ADM1**	24 hours	0.311±0.03	9
	84 hours	0.95±0.2	21
	One week	2.88±0.29	10
**ADM2**	24 hours	0.244±0.01	4
	84 hours	0.92±0.15	16
	One week	2.13±0.24	11

According to ANOVA, at 24 hours, cell viability was significantly different among the three groups (P=0.01). Post hoc tests showed no significant difference between the control and ADM1 groups (P>0.05); however, ADM2 was significantly different from the ADM1 and control groups (P<0.01).

At 84 hours, no significant difference was noted in cell viability among the three groups (P=0.4). At one week, cell viability in the control group was 1.7 times the rate in the ADM2 group and 22.7% higher than that in the ADM1 group. According to ANOVA, the differences in this regard were significant among the three groups (P=0.01). Pairwise comparisons showed that cell viability in the ADM1 group was higher than that in the ADM2 group. Also, cell viability in the control group was higher than that in the ADM1 and ADM2 groups (P<0.005).

The highest coefficient of variation was noted in the ADM1 group at 84 hours (21), followed by the ADM2 group (16).

### Cell Morphology

**Figure 3:**
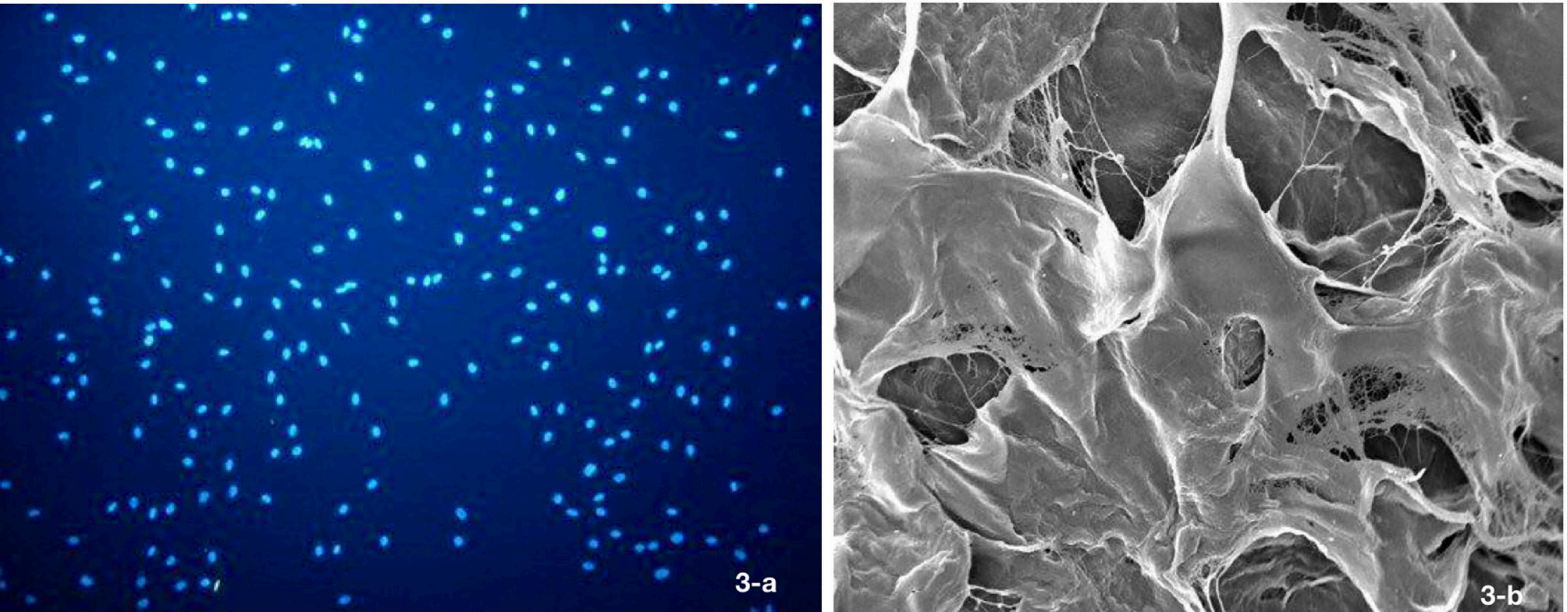
Electron microscopic micrograph of human fibroblasts on ADM1 (A) and ADM2 (B) scaffolds at 24 hours after culture at ×1000 magnification.

The assessment of cell morphology under SEM at 24 hours showed higher cell appendages in the ADM1 group compared to the ADM2 group (Figure 3).

## Discussion

This study assessed the effect of two commonly used ADMs on the attachment and viability of HGFs. DAPI and MTT assays were used to assess cell attachment. The results showed no significant difference between the two scaffolds in this respect. The assessment of cell viability with the MTT assay at 24 and 84 hours and seven days showed that viability in all the three groups increased over time, and at seven days, the viability of cells on ADM1 was significantly higher than that on ADM2. SEM assessment revealed that cells on ADM1 had more cellular appendages than those on ADM2.

Ghasemi et al^15^ assessed the adhesion of fibroblasts to three non-resorbable membranes (TXT-200, GBR-200, and IMTEC OVID) using the alkaline phosphatase test and MTT assay. They also assessed cell morphology. They showed that adhesion was higher in GBR-200 and TXT-200 in both tests but not significantly. However, morphologically, the attachment was significantly higher in these two groups. The membranes used in their study were non-resorbable, and due to their toxicity, cell adhesion was lower compared to resorbable membranes used in the present study. Also, the alkaline phosphatase test is not suitable for the assessment of cell attachment. Takta et al^16^ assessed the adhesion of rabbit fibroblasts to resorbable Tissue Guide and BioMend membranes in comparison with Goretex non-resorbable membrane and showed that adhesion and viability of fibroblasts on resorbable membranes were higher than those on the non-resorbable membrane. Their study was different from the present study in that they assessed cell adhesion using the alkaline phosphatase test and light microscopy, and the fibroblasts were isolated from rabbits. In studies by Ghasemi et al^15^ and Takta et al,^16^ the main factor causing ideal adhesion was the biocompatibility of membranes. However, factors such as orientation and thickness of fibers, membrane porosities, and composition of scaffolds can also affect adhesion and viability of fibroblasts.^1,15^ According to Schor,^17^ growth of fibroblasts on the collagen matrix is affected by factors such as the availability of nutrients, which limits the migration and growth of cells on the surface of the scaffold. Murphy et al^18^ showed that the size of pores significantly affected the attachment and viability of cells and demonstrated that the attachment and viability of cells on scaffolds with a mean pore size of 325 µ were significantly higher than those on scaffolds with smaller pores. A balance should be maintained between the size and number of pores since a small size of pores limits the availability of nutrients and migration of cells while a large size of pores decreases the surface area of the scaffold and consequently limits cell attachment.

Aside from the composition of the scaffold and the duration of cell culture, the cell type also affects the scaffold’s distribution in the clinical setting.^19^ Murphy et al^18^ used osteoblasts in their study. Hillman et al^19^ evaluated the attachment and viability of HGFs on Bio-Gide and Ethisorb resorbable membranes. After four weeks of cell culture, they noticed that HGFs had a better and more uniform distribution on the membrane surface with a looser collagen network than the membrane with a dense collagen network. However, the density of the collagen network did not affect cell morphology. They assessed these parameters using polymerase chain reaction and SEM, which were different from methods of assessment in the present study.

Maia et al^20^ cultured HGFs, canine gingival fibroblasts (CGF), and murine cancer cells on ADM for 14 days. Using epifluorescence, they showed that all cell lines spread on the ADM with low density at 12 hours. At seven and 14 days, CGFs distributed on ADM as a non-homogenous cell line, while HGFs formed a uniform homogenous cell layer on the scaffold. CGF counts did not increase during the study, while HGF and murine cancer cell counts significantly increased during the study period. The MTT assay showed that HGFs had higher viability than CGFs. Assessment of cell morphology by direct fluorescence at 12 hours showed polyclonal cells with round nuclei. At seven and 14 days, HGFs were elongated and spindle-shaped and showed higher activity, which was in agreement with SEM observations of ADM1 in the present study. Since in the study by Maia et al^20^ and Hillman et al,^19^ uniform distribution and attachment of cells on the scaffold surface occurred and the cell counts in deeper layers were low, it seems that selection of a scaffold with a lower density of collagen fibers results in more uniform attachment and distribution of cells on the scaffold surface. This may also explain the higher viability and proliferation of HGFs on the surface of ADM1.

Rodrigues et al^21^ showed that 90% of the adhesion of HGFs occurred seven days after culture on the surface of the scaffold. This result was in line with our findings since maximum cell attachment also occurred at seven days after culture in our study. Since a shorter recovery period is always preferred clinically, the time of maximum cell attachment to scaffolds is clinically important. However, Maia et al^20^ showed that 14 days of culture was the ideal time for the growth of fibroblasts on the surface of the scaffold.

This study had several advantages. We assessed both the attachment and viability of HGFs, which are essential parameters in wound healing and repair. A literature search yielded no study on cell attachment using both DAPI and MTT assay, which was a strength of our study. Also, no previous study was found to have compared these two scaffolds. The study was performed blindly, and each measurement was repeated five times. Empty wells were considered as the control group in our study, which are ideal for cell attachment and viability.

This study had an in vitro design. Thus, a generalization of results to the clinical setting should be made with caution. Further in vivo studies are required to compare the efficacy of these scaffolds.

## Conclusion

Both types of scaffolds were the same in terms of attachment of fibroblasts. Proliferation and viability of fibroblasts on ADM1 were significantly higher than those on ADM2 at seven days. HGFs on ADM1 were morphologically more active, showed greater distribution, and had more cellular appendages. ADM1 appears to be superior to ADM2 in terms of cell attachment, proliferation, viability, and morphology in vitro, indicating that the quality of acellular dermal scaffolds can significantly influence cellular behavior and tissue maturation.

## Authors’ Contributions

OM: Conceptualization, ESJ: Formal Analysis, BM and ESJ: Investigation, OM and BM: Methodology, BM and OM: Project Administration, BM and ESJ: Writing – Original Draft, OM and BM: Writing – Review & Editing. All authors have read and approved the final manuscript.

## Competing Interests

The authors declare that there were no competing interests between the authors.

## Ethical Approval

The study was conducted in an in-vitro environment and no humans participated in the study. The study did not have any ethical registrations.
